# Analysis of differences between early-onset and late-onset early gastric cancer treated with endoscopic submucosal dissection

**DOI:** 10.3389/fmed.2026.1884527

**Published:** 2026-08-18

**Authors:** Linzhen Li, Xuhua Xiao, Yan Zhang

**Affiliations:** 1 Department of Gastroenterology, First Affiliated Hospital of Wannan Medical College, Wuhu, Anhui Province, China; 2 Department of Gastroenterology, The Affiliated Hospital of Guilin Medical University, Xiufeng District, Guilin, China

**Keywords:** early-onset early gastric cancer, endoscopic submucosal dissection, late-onset early gastric cancer, pathological type, postoperative complications

## Abstract

**Background and purpose:**

Gastric cancer is one of the most common cancers worldwide, with high incidence and mortality rates. The aim of this study was to analyze the differences in demographics, lesion morphology, and pathology between early-onset and late-onset early gastric cancer.

**Patients and methods:**

A total of 37 early-onset early gastric cancer (EO-EGC) patients who underwent endoscopic submucosal dissection (ESD) were included in the study. For comparison, 37 late-onset early gastric cancer (LO-EGC) patients who underwent ESD during the same period were randomly selected as the control group. We compared EO-EGC and LO-EGC with respect to lesion size, location, color, postoperative pathological type, depth of invasion, and postoperative complications.

**Results:**

The LO-EGC group had significantly more hypertensive patients (*P* = 0.030) and larger lesions (*P* = 0.025) than the EO-EGC group. Pathologically, high-grade intraepithelial neoplasia was predominant in the EO-EGC group, while differentiated tubular adenocarcinoma was the main type in the LO-EGC group (*P* < 0.01). Lesions were mainly in the antrum in the EO-EGC group, and evenly distributed in the antrum, incisura angularis, and cardia in the LO-EGC group (*P* = 0.092, no statistical significance). No significant differences were observed between the two groups in gender (*P* = 0.183), diabetes mellitus (*P* = 1.000), coronary artery disease (*P* = 0.615), current or prior Helicobacter pylori infection (*P* = 0.485), lesion color (*P* = 0.358), invasion depth (*P* = 0.613), delayed postoperative bleeding (*P* = 1.000), delayed postoperative perforation (*P* = 0.247), or the need for additional surgery (*P* = 0.057).

**Conclusions:**

EO-EGC and LO-EGC exhibit significant differences in hypertension comorbidity, lesion size and pathological type, while their lesion distribution and perioperative clinical outcomes remain similar.

## 1 Introduction

Gastric cancer (GC) ranks as the fifth most prevalent cancer globally, with notably high incidence and mortality (1, 2). The pathogenic mechanism of gastric cancer is intricate, with its development driven by the combined contributions of environmental, genetic and microbial components. Among these, Helicobacter pylori (HP) is a major risk factor for gastric cancer, and numerous studies have confirmed its crucial role in the initiation and progression of gastric cancer (3–5). Early-onset gastric cancer (EOGC) is defined as gastric cancer occurring in patients under the age of 50 years old, with unique epidemiological and pathological characteristics (6–8). Studies have shown that smoking and a high-salt diet significantly increase the incidence of EOGC (9, 10).

Early gastric cancer (EGC) is defined as carcinoma confined to the mucosa or submucosa, regardless of lymph node metastasis. EGC has a low metastasis risk and high cure rate, with a 5-year survival rate exceeding 90% (11). According to the Japanese Gastric Cancer Association JGCA classification system, EGCs fall under subtype 0, which is further categorized into 0-I (protruding), 0-IIa (superficial elevated), 0-IIb (superficial flat), 0-IIc (superficial depressed) and 0-III (excavated) types (12). Endoscopic submucosal dissection (ESD) is currently the first-line treatment for early gastric cancer without lymph node metastasis (13, 14). Study findings demonstrate that ESD is significantly superior to EMR in terms of en bloc resection rate, curative resection rate, and local recurrence rate (15). In experienced high-volume centers, this approach yields a curative resection rate exceeding 95%, with detailed histopathological assessment of invasion depth, differentiation, and vascular involvement available (16). The main common complications after ESD are delayed bleeding and delayed perforation, with an overall incidence typically < 5%. However, curative ESD fails to eliminate Helicobacter pylori-associated chronic atrophic gastritis and intestinal metaplasia; thus, 2.8% to 15.9% of patients will develop metachronous EGC within 10 years, defined as a new carcinoma occurring ≥12 months after index resection (17).

Although numerous studies have investigated the incidence and mortality of EOGC, few have focused on EOGC treated with ESD. Therefore, the aim of this study was to evaluate the differences in demographics, lesion morphology, and pathology between early-onset early gastric cancer (EO-EGC) and late-onset early gastric cancer (LO-EGC).

## 2 Methods

### 2.1 Ethical considerations

The research was performed according to the Declaration of Helsinki including patients' consent. The study was approved by the Institutional Review Board of Wannan Medical College (No. 19, 2024). All participants give informed consent.

### 2.2 Patients and study design

**Figure 1:**
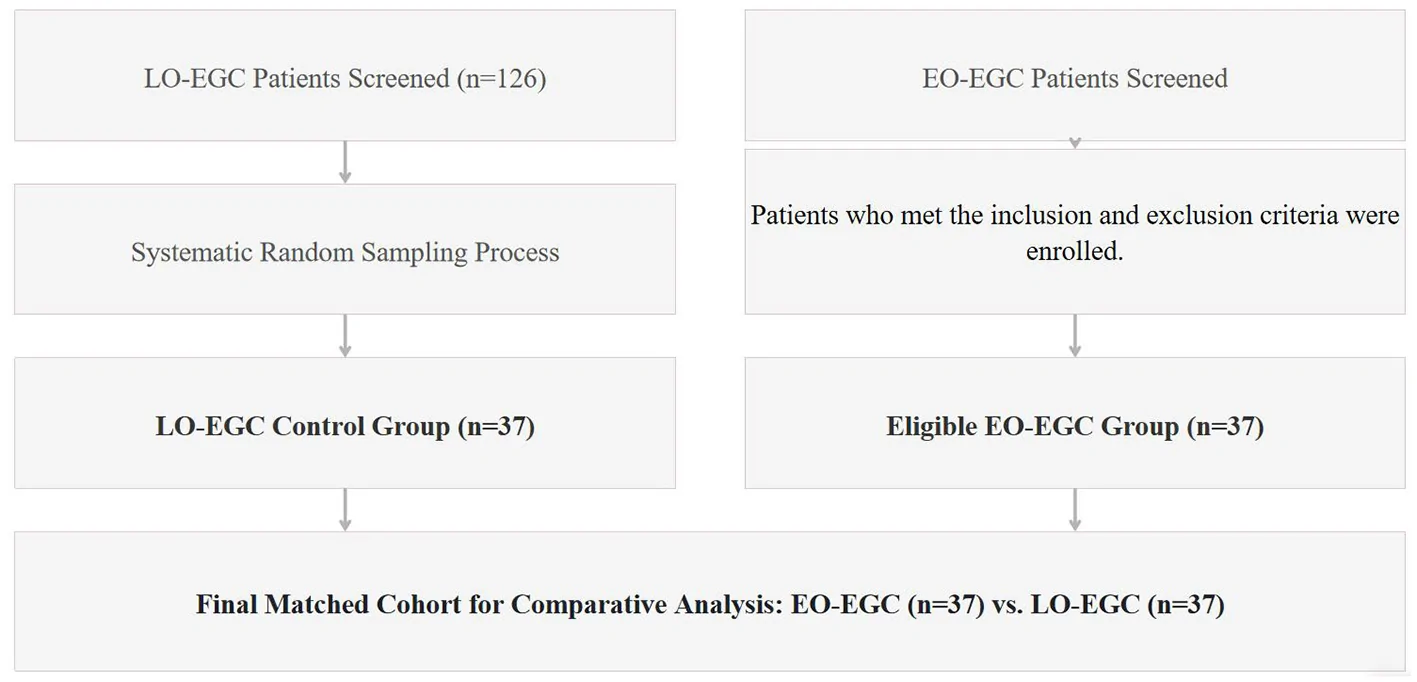
Flowchart of participant enrollment.

In this multicenter retrospective study, a total of 37 EO-EGC patients were evaluated (March 2020–December 2025). The inclusion criteria were as follows: (1) Patients whose post-ESD pathological results indicated early-stage gastric cancer; (2) the patient's basic information and the details of the lesion have been recorded completely. The exclusion criteria were as follows: (1) the postoperative pathology confirmed that it was not an early-stage gastric cancer; (2) the records of postoperative complications are not detailed enough. Record the basic information of all patients, including gender, age, and histories of hypertension, diabetes mellitus, coronary heart disease, and current or prior Helicobacter pylori infection. The lesion information of all patients before ESD was also recorded, including lesion location, lesion size, Paris endoscopic classification (16), and lesion color. Postoperative pathological type, invasion depth, postoperative complications (delayed bleeding and delayed perforation), need for additional surgery, and occurrence of metachronous carcinoma were recorded for all patients. This study adopted the diagnostic criteria proposed by the Japanese Gastric Cancer Association (JGCA). We randomly selected 37 patients with LO-EGC as controls from 126 eligible LO-EGC patients. Specifically, all 126 patients were randomly reordered, and one subject was enrolled at an interval of every two patients. The same parameters were also documented. Differences in all the above parameters between the EO-EGC and LO-EGC groups were compared (Figure 1).

### 2.3 Statistical analysis

Descriptive statistics were expressed as median (interquartile range) or percentage. All continuous variables were first assessed for normal distribution. The Mann-Whitney U test was applied for comparisons of nonparametric variables, while categorical variables were analyzed using the chi-square test or Fisher's exact test. All statistical analyses were conducted with SPSS 27.0 software, and a *P* value < 0.05 was regarded as statistically significant.

## 3 Results

### 3.1 General characteristics and lesion-related information of EO-EGC

A total of 37 EO-EGC patients were included in this study, comprising 25 males and 12 females. The median age (interquartile range) was 45 (40–49) years. Nine patients (24.32%) had hypertension, two (5.4 1%) had diabetes mellitus, and one (2.70%) had coronary artery disease. The tumor was located at or below the cardia in four patients (10.81%), in the fundus in three (8.11%), in the body in six (16.22%), at the angle in six (16.22%), and in the antrum in 18 (48.65%). The median lesion size (interquartile range) was 12 (9–20) mm. The lesion was erythematous in 36 patients (97.30%) and pale in only 1 patient (2.70%). Postoperative pathology revealed high-grade intraepithelial neoplasia in 24 patients (64.86%), differentiated tubular adenocarcinoma in 8 patients (21.62%) (including 2 well-differentiated and 6 moderately differentiated adenocarcinomas), and undifferentiated adenocarcinoma in 5 patients (13.51%) (including 2 poorly differentiated adenocarcinomas and 3 signet ring cell carcinomas). Postoperative pathology showed that the depth of invasion was T1a in 11 patients and T1b in 2 patients. Two patients experienced delayed postoperative bleeding. There were no cases of delayed postoperative perforation or additional surgery (Table 1).

**Table 1:** Clinical features of EO-EGC patients.

Sex: male, *n* (%)	25 (67.57%)
Age (years): M (QR)	45 (40–49)
Hypertension: *n* (%)	9 (24.32%)
Diabetes mellitus: *n* (%)	2 (5.41%)
Coronary artery disease: *n* (%)	1 (2.70%)
Current or prior Helicobacter pylori infection	17 (45.95%)
Lesion location	
Cardia and subcardial region: *n* (%)	4 (10.81%)
Fundus: *n* (%)	3 (8.11%)
Body: *n* (%)	6 (16.22%)
Gastric angle: *n* (%)	6 (16.22%)
Antrum: *n* (%)	18 (48.65%)
Lesion size: M (QR)	12 (9–20)
Lesion color	
Erythema: *n* (%)	36 (97.30%)
Fading: *n* (%)	1 (2.70%)
Postoperative pathology	
High-grade intraepithelial neoplasia: *n* (%)	24 (64.86%)
Differentiated carcinoma: *n* (%)	8 (21.62%)
Undifferentiated carcinoma: *n* (%)	5 (13.51%)
Depth of invasion	
T1a: *n* (%)	11 (84.62%)
T1b: *n* (%)	2 (15.38%)
Postoperative delayed bleeding: *n* (%)	2 (5.41%)
Postoperative delayed perforation: *n* (%)	0
Additional surgery: *n* (%)	0

EO-EGC, early-onset early gastric cancer; M, median; QR, Quartile Range.

### 3.2 Analysis of the differences of parameters between EO-EGC group and LO-EGC group

The number of patients with hypertension (*P* = 0.030) was significantly higher in the LO-EGC group than in the EO-EGC group, with a statistically significant difference. There were no significant differences between the two groups in terms of gender (*P* = 0.183), the number of patients with diabetes mellitus (*P* = 1.000), the number of patients with coronary artery disease (*P* = 0.615), or current or prior Helicobacter pylori infection (*P* = 0.485). In the EO-EGC group, lesions were mainly located in the antrum, whereas in the LO-EGC group, the numbers of lesions located in the antrum, incisura angularis, and cardia were similar, although the difference was not statistically significant (*P* = 0.092). The lesion size was significantly larger in the LO-EGC group than in the EO-EGC group, with a statistically significant difference (*P* = 0.025). In the EO-EGC group, postoperative pathology was dominated by high-grade intraepithelial neoplasia, whereas in the LO-EGC group, differentiated tubular adenocarcinoma was the predominant pathological type (*P* < 0.01). There were no statistically significant differences between the two groups in terms of lesion color (*P* = 0.358), depth of invasion (*P* = 0.613), delayed postoperative bleeding (*P* = 1.000), delayed postoperative perforation (*P* = 0.247), or the need for additional surgery (*P* = 0.057) (Table 2).

**Table 2:** Analysis of the differences between EO-EGC group and LO-EGC group.

Parameters	EO-EGC group *n* = 37	LO-EGC group *n* = 37	*P* value
Sex: male, *n* (%)	25 (67.57%)	30 (81.08%)	0.183
Hypertension: *n* (%)	9 (24.32%)	18 (48.65%)	0.030
Diabetes mellitus: *n* (%)	2 (5.41%)	3 (8.11%)	1.000
Coronary artery disease: *n* (%)	1 (2.70%)	3 (8.11%)	0.615
Current or prior Helicobacter pylori infection	17 (45.95%)	20 (54.05%)	0.485
Lesion location:			0.092
Cardia and subcardial region: *n* (%)	4 (10.81%)	11 (29.73%)	
Fundus: *n* (%)	3 (8.11%)	0	
Body: *n* (%)	6 (16.22%)	5 (13.51%)	
Gastric angle: *n* (%)	6 (16.22%)	9 (24.32%)	
Antrum: *n* (%)	18 (48.65%)	12 (32.43%)	
Lesion size: M (QR)	12 (9–20)	18 (14–25)	0.025
Lesion color:			0.358
Erythema: *n* (%)	36 (97.30%)	33 (89.19%)	
Fading: *n* (%)	1 (2.70%)	4 (10.81%)	
Postoperative pathology:			< 0.01
High-grade intraepithelial neoplasia: *n* (%)	24 (64.86%)	11 (29.73%)	
Differentiated carcinoma: *n* (%)	8 (21.62%)	25 (67.57%)	
Undifferentiated carcinoma: *n* (%)	5 (13.51%)	1 (2.70%)	
Depth of invasion:			0.613
T1a: *n* (%)	11 (84.62%)	21 (80.77%)	
T1b: *n* (%)	2 (15.38%)	5 (19.23%)	
Postoperative delayed bleeding: *n* (%)	2 (5.41%)	3 (8.11%)	1.000
Postoperative delayed perforation: *n* (%)	0	2 (5.41%)	0.247
Additional surgery: *n* (%)	0	4 (10.81%)	0.057

EO-EGC, early-onset early gastric cancer; LO-EGC, late-onset early gastric cancer; M, median; QR, Quartile Range.

## 4 Discussion

In recent years, the incidence of EOGC has been on the rise (18). In this study, EOGC was defined as occurring in patients younger than 50 years of age, although other studies have used cutoffs of 40 or 45 years (19–22). The exact reasons for the increasing incidence of EOGC remain unclear, but may be related to dietary factors, genetic susceptibility, gastric microbiota dysbiosis, and screening of high-risk individuals (23). The clinicopathological characteristics of EOGC differ significantly from those of LOGC, especially with respect to gender, histological type, cell differentiation, and pathological stage. Previous studies (24, 25) have confirmed that the proportion of female patients with EOGC is as high as 45%, whereas in LOGC it is only about 30%. In the present study, the proportion of female patients was 32.43% in the EOGC group and 19.92% in the LOGC group, but the difference was not statistically significant. The absence of a statistically significant difference may be attributable to the limited sample size. In the present study, we found that in terms of cell differentiation, the proportion of undifferentiated carcinomas (including poorly differentiated adenocarcinoma and signet ring cell carcinoma) was significantly higher in EO-EGC than in LO-EGC. This finding is consistent with previously published results regarding advanced gastric cancer (25, 26). Whether in early-stage or advanced gastric cancer, the proportion of poorly differentiated tumors is higher in EOGC compared with LOGC. Therefore, compared with LOGC, EOGC exhibits a higher degree of malignancy in its clinicopathological characteristics. Thus, greater attention should be paid to the screening and early diagnosis of EOGC to improve its treatment rate and prognosis. All 37 patients with EO-EGC in the present study were successfully treated with ESD alone without additional surgery. This indicates that early detection of EOGC can help avoid partial or total gastrectomy, resulting in substantial benefits for patients.

The present study found that the majority of EO-EGC were located in the antrum, accounting for nearly 50%, whereas in LO-EGC, the cardia and subcardia, gastric angle, and antrum were affected with similar frequencies. Several factors may explain this difference. First, HP infection in younger patients more commonly affects the antrum, leading to localized chronic inflammation and increasing cancer risk in this region. In contrast, older patients often develop extensive mucosal atrophy and intestinal metaplasia, resulting in a more dispersed pattern of carcinogenesis. Second, EO-EGC is more frequently of an undifferentiated type, which tends to arise in the antrum, whereas LO-EGC is often intestinal-type and associated with widespread mucosal damage. Finally, age-related physiological changes, including the progression of multifocal atrophy from the antrum to the corpus, contribute to the more uniform distribution of LO-GGC. This study also found that the lesions of EO-EGC were significantly smaller than those of LO-EGC. This may be because older patients often undergo examination only after symptoms have persisted for a period of time, resulting in larger lesions. In contrast, younger patients are more likely to be diagnosed during routine health checkups when the disease is at an earlier stage. This also explains why all 37 cases of EO-EGC were treated with curative ESD, whereas 4 of the 37 cases of LO-EGC required additional surgery due to deeper submucosal invasion.

Several limitations of the present study should be acknowledged. First, this single-center retrospective analysis with a relatively small sample size may compromise the generalizability and reliability of our findings. We did not perform *a priori* sample size calculation before patient enrollment, which is a notable limitation of this retrospective observational study. Second, inherent selection bias exists because our cohort only included patients receiving ESD, a highly selected sample that cannot fully represent all early gastric cancer patients. In addition, a considerable proportion of early-onset early gastric cancer lesions were high-grade intraepithelial neoplasia, which cannot be stratified according to histological differentiation status. Finally, long-term survival and recurrence follow-up data were unavailable; thus, we failed to analyze the correlation between clinicopathological features and long-term clinical outcomes.

In conclusion, patients with EO-EGC tend to have poorer histologic differentiation, and tend to occur in the gastric antrum. The lesions are smaller compared with those of LO-EGC, and their color is mainly erythematous.

## Data Availability

The original contributions presented in the study are included in the article/supplementary material, further inquiries can be directed to the corresponding author.
